# Extract from Private Letter

**Published:** 1877-10-20

**Authors:** 


### EXTRACT FROM PRIVATE LETTER.

"The remarks contained in your last issue deprecating the low state of medical education was freely talked of in our Society. The opinion seemed to be general that the Record ought to be in the hands of every intelligent physician in the South, and that the members of the profession, as preceptors, and in their organized capacity as Societies, etc., should observe the sentiments upon the subject of education referred to, and that pupils should be advised to attend first-class Colleges only." R. T. L——.

It always gratifies us to receive letters containing evidences of approval like the above. If every present subscriber will renew and send one or more new subscribers for the ensuing year, we will thereby be enabled so to improve the journal as to increase its popularity more and more, and would soon place it in the hands of all progressive and intelligent readers.

